# Mating-Related Traumatic Lesions in Slow-Growing Broiler Breeder Hens: Morphological and Physiological Correlates

**DOI:** 10.3390/vetsci13101115

**Published:** 2026-10-19

**Authors:** Mihnea Lupu, Raluca Ioana Rizac, Nicolae Tiberiu Constantin, Florin Iordache, Manuella Militaru

**Affiliations:** Faculty of Veterinary Medicine, University of Agronomic Sciences and Veterinary Medicine of Bucharest, 105 Splaiul Independentei, District 5, 050097 Bucharest, Romania; mihnea.l96@gmail.com (M.L.); tiberiu.constantin@fmvb.usamv.ro (N.T.C.); manuella.militaru@fmvb.usamv.ro (M.M.)

**Keywords:** aggressive mating, breeder hen, hen ovarian ultrasonography, rooster-induced injury, serum corticosterone

## Abstract

In broiler breeder flocks, mating may cause traumatic skin lesions in hens, yet their biological significance remains insufficiently understood. This study aimed to investigate associations between mating-related lesions and physical and physiological status in broiler breeder hens, focusing on lesion severity and anatomical location. At 40 weeks, 39 hens were examined, including 29 with mating-related lesions and 10 lesion-free controls. Birds underwent clinical examination, body weight and plumage assessment, serum corticosterone measurement, and ultrasonographic evaluation of ovarian activity. Affected hens had lower body weight than controls, and 58.6% showed ovarian inactivity. Within affected hens, body weight was positively associated with plumage score and ovarian activity. Gross lesion severity was not consistently associated with body weight, plumage condition, or ovarian activity, whereas lesion location provided additional information on these indicators. Hens with cephalic lesions had lower body weight, poorer plumage condition, and lower ovarian activity than hens with trunk lesions, despite less severe lesions macroscopically. Serum corticosterone concentrations did not differ between groups or vary with lesion severity or location. These findings suggest that mating-related lesions may reflect more than superficial injury and that lesion location, particularly cephalic involvement, may provide useful information on the biological status of affected hens.

## Simple Summary

Mating-related injuries are a recurring welfare concern in broiler breeder hens, but their importance for the health and reproductive function of affected birds is not well understood. This study investigated whether hens with mating-related injuries differed from unaffected hens in body weight, feather condition, stress-related hormone levels, and ovarian activity, assessed using ultrasound examination. Injured hens had lower body weight than unaffected hens, and 58.6% showed no detectable ovarian activity. Among injured hens, lower body weight was associated with poorer feather condition and lower ovarian activity. The location of the injury, particularly involvement of the head, was a better indicator of the overall condition of affected hens than the visible severity of the injury. These findings suggest that mating-related injuries may be associated with broader changes in hen health and reproductive status and that their location may be useful when assessing affected birds. Recognizing these injuries, particularly those involving the head, may help farmers and veterinarians identify hens requiring closer monitoring and improve welfare assessment in breeder flocks. Further studies following hens over time are needed to determine whether injuries contribute to declining condition and reproductive function or whether hens in poorer condition are more likely to become injured.

## 1. Introduction

In broiler breeder flocks, mating may result in traumatic skin lesions in hens, particularly when male sexual behavior is rough or forced. These traumatic lesions, typically affecting the dorsal head region, back, flanks, and areas beneath the wings, have been reported for decades and are recognized as a recurring problem in commercial breeder systems [1,2,3,4,5,6,7,8,9]. In addition to their practical implications for flock management, mating-related injuries are increasingly regarded as a welfare concern because they may compromise physical integrity, expose hens to repeated tissue damage, and contribute to chronic stress [5,6,10].

Previous studies have addressed this issue mainly from a behavioral perspective. Research in broiler breeders has focused primarily on male sexual behavior, mating aggressiveness, and the effects of factors such as genotype, feeding regimen, or sexual experience on mating interactions [2,3,4,5,6,7,11]. Some studies also examined female receptivity, mate preference, and avoidance behavior, showing that hens are not passive participants in the mating interaction [2]. However, even when hens were included, the main emphasis remained on the causes and behavioral expression of mating aggressiveness, rather than on the consequences of traumatic lesions for the female.

Although lesions in hens have been repeatedly mentioned in the literature, they have usually been described only in general terms or as part of broader flock-level problems, including reduced fertility, increased mortality, and impaired welfare [4,5,6]. Earlier pathological descriptions documented the location and severity of such lesions, especially in the occipital region, flanks, and thighs, and strongly suggest an association with mating activity, even when they were not explicitly interpreted in the context of aggressive male behavior against females [1]. Nevertheless, limited information is available on the biological significance of these lesions for hens, particularly from an integrated morphological and physiological perspective.

This limitation in the current literature is relevant because mating-related traumatic lesions may reflect more than mere external tissue damage. They may be associated with alterations in physical status and the activation of stress-related physiological responses, all of which are relevant to hens’ condition and, potentially, to animal welfare [12,13,14,15]. From a physiological perspective, repeated injury and persistent exposure to stressful mating interactions may trigger a stress response, leading to increased corticosterone secretion. Although Válková et al. (2023) examined traumatic limb injuries associated with handling and transport rather than mating-related lesions, their findings support the view that traumatic injury in hens is associated with increased stress [16]. In birds, elevated corticosterone is widely recognized as an indicator of stress [17]. In addition, stress has been shown to impair ovarian function and reproductive performance [18,19]. Moreover, exogenous glucocorticoid administration has been shown to significantly decrease laying performance in hens [20]. Therefore, evaluating corticosterone alongside ovarian status may provide a more comprehensive understanding of how mating-related traumatic lesions affect hens and their potential consequences for reproductive function. This question is particularly relevant under commercial conditions, where genotype and management practices may influence health, behaviour, welfare, and reproductive performance [21]. It also remains insufficiently explored in slow-growing breeder populations compared with conventional broiler breeder lines. Therefore, building on our previous observations on the temporal occurrence of mating-related traumatic lesions [9], the present study focused on the biological status of affected hens under commercial conditions. Specifically, we compared affected and unaffected slow-growing broiler breeder hens raised under the same conditions through an integrative assessment of body weight, plumage condition, serum corticosterone concentration, and ultrasonographically detectable ovarian activity. The aim was to determine whether mating-related traumatic lesions were associated with alterations in hens’ physical and physiological status, with potential implications for welfare and reproductive status.

## 2. Materials and Methods

### 2.1. Animals and Study Design

This study was conducted in a commercial breeder flock comprising 4672 hens from the parent stock of a slow-growing colored broiler hybrid. At 40 weeks of age, 39 hens from this flock were enrolled in the study, including 29 affected birds randomly selected from hens with visible traumatic lesions and 10 lesion-free controls selected according to predefined eligibility criteria (intact plumage, good general clinical condition, and absence of traumatic lesions or other findings compatible with mating-related trauma). Accordingly, the affected group represented a sampled subset of injured hens present at the time of evaluation rather than the entire affected population.

Affected hens were identified according to a predefined case definition based on the anatomical distribution and gross morphology of lesions consistent with mating-associated trauma, as described in the literature, particularly lesions affecting the dorsal head region, back, flanks, and areas beneath the wings [1,4] and characterized by skin discontinuity, necrosis, and purulent changes [1], and supported by the authors’ previous research and field observations [8,9]. Lesions suggestive of alternative etiologies were excluded from case classification, and birds with unrelated clinical abnormalities were not included. All enrolled hens originated from the same flock and were maintained under identical environmental and management conditions. Birds were housed on floor litter in a closed house under artificial lighting, with wood-shavings litter and a stocking density of 8 females/m^2^. The male-to-female ratio was 9:100. Males had undergone standard beak trimming and rear toe claw trimming to reduce the risk of mating-associated injuries; however, their spurs were present and were not trimmed or removed.

The hens were maintained in the same house from one day of age. At 21 weeks of age, the hens were photo-stimulated by increasing the light intensity from 5 to 20 lux and the photoperiod from 10 to 12 h of light per day. At the same age, roosters were introduced into the hens’ housing unit. Egg production commenced at approximately 21 weeks of age, when the first eggs were recorded. At 40 weeks of age, hens received 120 g of feed per hen per day using an automatic oval pan feeding system (KiXoo^®^, Roxell, Maldegem, Belgium). The daily feed allowance was provided in two meals, with 60% offered at 05:00 and 40% offered at 13:00.

### 2.2. Clinical Assessment and Lesion Index

Traumatic lesions were assessed by direct visual inspection and palpation. All hens were examined by the same evaluator during a single assessment session. The entire body surface was inspected, and the lesion location, the number of traumatic lesions per hen, lesion size, and signs of tissue repair were recorded. All cases were photographically documented at the time of assessment.

For each visible lesion, the 2 largest perpendicular dimensions were measured with a caliper and recorded as maximal length (L) and width (W). The lesion area was estimated using the ellipse formula (A = π × L × W/4), following an approach adapted from Giggin et al. (2024) [22]. For prominent abscess-like lesions in the cephalic region, the estimated area was defined as the projected base area on the skin surface, allowing for comparison across lesion types. Multiple lesions were not observed in the hens included in this study; had they been present, individual lesion areas would have been summed for index calculation.

A study-specific composite lesion index was constructed to integrate lesion extent and reparative status into a single ordinal variable for comparative and correlational analyses with other study variables. Based on the distribution of lesion area in affected hens, quartile-derived thresholds were used to define four lesion-size categories: 0 for lesions > Q3, 1 for lesions > Q2 to ≤Q3, 2 for lesions > Q1 to ≤Q2, and 3 for lesions ≤ Q1, where Q1 = 6.59 cm^2^, Q2 = 12.57 cm^2^, and Q3 = 19.84 cm^2^. One additional point was assigned when reparative tissue responses were present, including granulation tissue formation or marginal re-epithelialization. No additional point was assigned in the absence of reparative changes or in the presence of necrotic or purulent changes.

The final lesion index was calculated as the sum of the size and tissue-response categories, with a maximum value of 4. Higher lesion index values indicated a more favorable lesion status, characterized by less extensive lesions and more advanced reparative changes, whereas lower values indicated more extensive and clinically less favorable lesions.

### 2.3. Plumage Assessment

Plumage condition was evaluated by visual inspection by the same examiner during the same session as lesion assessment. Hens were carefully restrained in an upright position to allow for examination of the dorsal, lateral, and caudal body regions, and photographic documentation was obtained for each bird.

A global 6-point ordinal plumage score (0 to 5) was assigned based on overall plumage coverage and appearance, without region-specific scoring. Feather loss directly attributable to traumatic lesions was excluded to avoid overestimation of generalized plumage deterioration. Scores were defined as follows: 5, complete, dense, and uniform plumage, with no visible deterioration; 4, absence of contour feathers over limited areas in the dorsocaudal trunk region and, occasionally, on the dorsolateral wing surfaces, with down and small contour feathers still present such that the skin remained unexposed; 3, loss of contour feathers over extensive areas of the dorsal and dorsolateral regions, with focal skin exposure, while small contour feathers and down were still partially present but did not provide complete coverage; 2, extensive and clearly visible areas of exposed skin, particularly in the dorsocaudal trunk region, head, and dorsolateral wing surfaces; 1, feather loss extending beyond the previously described regions to include the dorsal thoracic region, with marked skin exposure and only sparse, unevenly distributed residual down or small contour feathers; and 0, absence of plumage over the dorsal head, dorsal cervical region, dorsal trunk from the cervical region to the tail, and dorsolateral wing surfaces.

### 2.4. Body Weight Determination

Body weight was measured individually in all study hens using an electronic poultry scale (BAT 1, VEIT Electronics, Moravany, Czech Republic; SN: 110731110). Body weight values were recorded in grams and used for subsequent comparative and correlational analyses.

### 2.5. Ultrasonographic Assessment of Ovarian Activity

Ovarian ultrasonography was used as a noninvasive method to assess reproductive status. The examination recorded the presence of visible ovarian follicles, the number of detectable follicles, the maximal diameter of the largest follicle, and the absence of detectable follicular structures, interpreted as ovarian inactivity.

**Figure 1:**
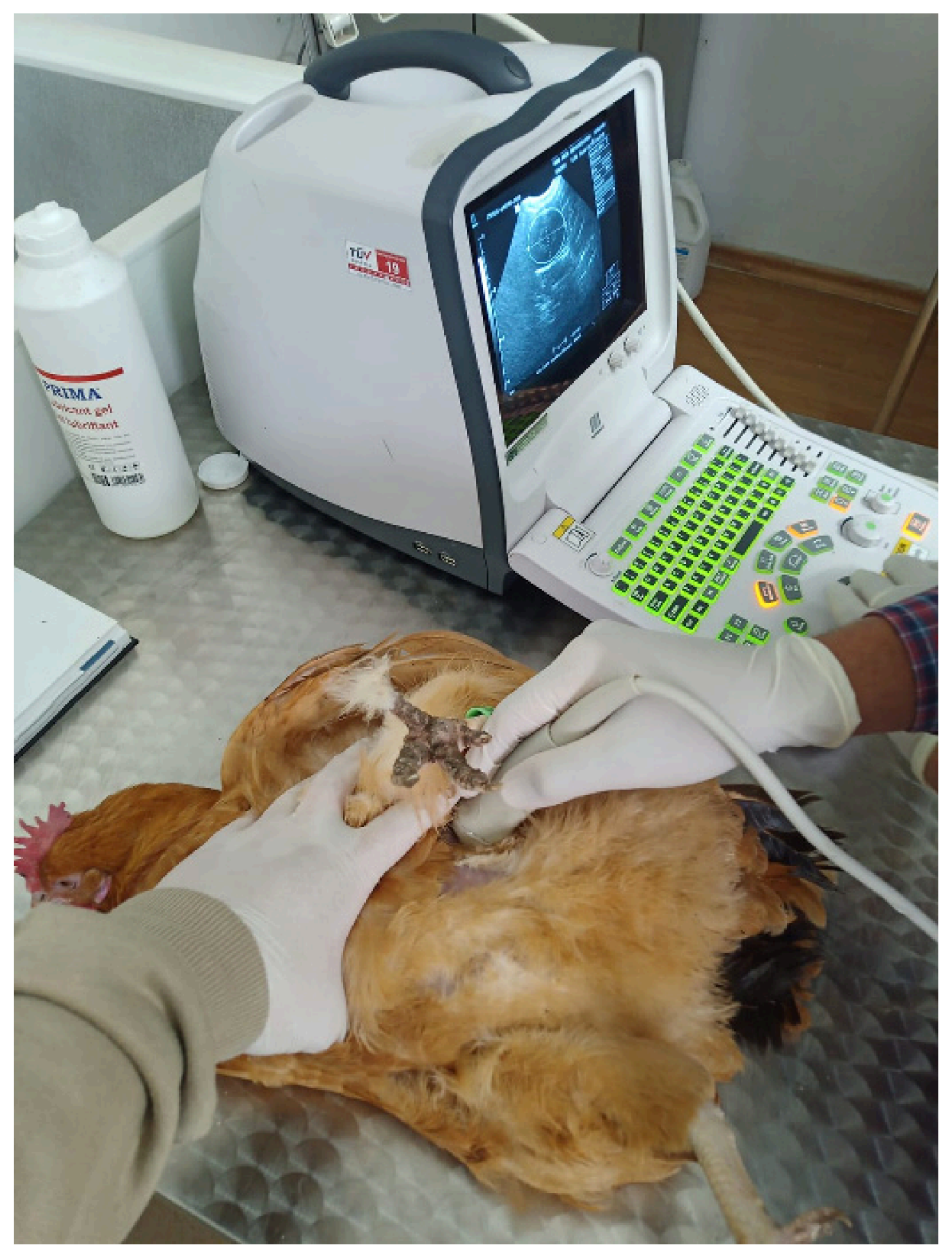
Ultrasound examination of the ovary in the hen: technique and positioning.

Ultrasonographic examination was performed using a portable ultrasound device (Mindray DigiPrince DP-6900 Vet, Shenzhen Mindray Bio-Medical Electronics Co., Ltd., Shenzhen, China) equipped with a 3.5–7 MHz microconvex transducer (Shenzhen Mindray Bio-Medical Electronics Co., Ltd., Shenzhen, China). Bird positioning was adapted from Fajri and Ulum (2019) [23] with modifications for field conditions. Hens were examined in right lateral recumbency, with the left pelvic limb elevated craniolaterally to expose the left abdominal region (Figure 1). The transducer was placed between the last rib and the iliac margin. All examinations were performed transcutaneously, without feather clipping, using ultrasound gel, and were conducted by the same operator.

Ovarian status was classified as active when one or more follicles were identified and as inactive when no follicular structures were detected.

A study-specific ovarian activity score was constructed for affected hens using ultrasonographic findings in control hens as an internal reference framework. The control hens were used for this purpose because they belonged to the same genetic line, were of the same age, were maintained under identical husbandry conditions, and had been selected for good overall clinical condition. The score comprised a follicular diameter subscore and a follicle number subscore. For follicular diameter, the threshold for the highest category was set at 33.9 mm, corresponding to the minimum value observed in the control hens. An additional threshold at 20.0 mm was introduced empirically to distinguish markedly reduced follicular development from values approaching the control range. Follicle number categories were defined pragmatically to reflect increasing ovarian activity. The diameter subscore was defined as follows: 0, no follicles detected; 1, maximal follicular diameter <20.0 mm; 2, maximal follicular diameter 20.0 to <33.9 mm; and 3, ≥33.9 mm. The follicle number subscore was defined as follows: 0, no follicles detected; 1, 1 follicle; 2, 2 to 3 follicles; and 3, ≥4 follicles. The total ovarian activity score was calculated as the sum of both subscores, yielding values from 0 to 6, with higher scores indicating greater ultrasonographic evidence of ovarian activity.

### 2.6. Blood Sampling, Serum Processing, and Corticosterone Assay

Blood samples were collected from all hens beginning at 09:30. Following individual restraint, 2 mL of blood was obtained from the brachial vein using a 21-gauge butterfly needle, with a mean sampling time of approximately 1.5 min from initial capture. Samples were collected into blood collection tubes containing clot activator and separation gel, allowed to clot for 30 min at room temperature (approximately 24 °C), centrifuged at 300× *g* for 20 min, and separated serum was transferred into microtubes and stored at −20 °C until analysis.

Serum corticosterone was measured using a competitive ELISA kit validated for avian samples (Chicken Corticosterone ELISA Kit, ELK Biotechnology, Wuhan, China; catalog no. ELK8925; lot no. 17462135; 48-test format) [24], according to the manufacturer’s instructions. Samples were analyzed in single wells. The reported assay sensitivity was 0.5 ng/mL, the detection range was 1.57 to 100 ng/mL, and intra- and inter-assay coefficients of variation were <10%. Absorbance was read at 450 nm with a BIO-RAD Model 680 microplate reader (Bio-Rad Laboratories, Inc., Hercules, CA, USA), and concentrations were derived from the standard curve generated with the supplied standards [25].

### 2.7. Procedural Sequence

Animal-based study procedures were performed over 3 consecutive days. On day 1, hens were selected, individually identified with leg rings, and examined by ovarian ultrasonography. On day 2, blood samples were collected. On day 3, body weight, plumage condition, and traumatic lesions were assessed. The sequence was standardized across all birds to minimize procedure-related variation, particularly with respect to serum corticosterone concentration.

### 2.8. Statistical Analysis

Statistical analyses were performed using Jamovi (version 2.7.4; The Jamovi Project, Sydney, Australia), an open-source software package based on the R programming language. Microsoft Excel 2016 (Microsoft Corp., Redmond, WA, USA) was used for data organization and preliminary processing. Given the small sample size and the ordinal or non-normally distributed nature of several variables, nonparametric methods were used throughout. Differences between control and affected hens, as well as between lesion-location categories within the affected group, were assessed using the Mann–Whitney U test. Each affected hen was assigned to a single lesion-location category (head or trunk). Comparisons between lesion-location categories were considered exploratory, given the small number of hens with cephalic lesions (*n* = 6). No adjustment for multiple testing was applied; therefore, the resulting *p* values should be interpreted cautiously. Associations between continuous or ordinal variables were evaluated using Spearman’s rank correlation coefficient. Statistical significance was set at *p* < 0.05.

## 3. Results

### 3.1. Lesion Characteristics in Affected Hens

**Figure 2:**
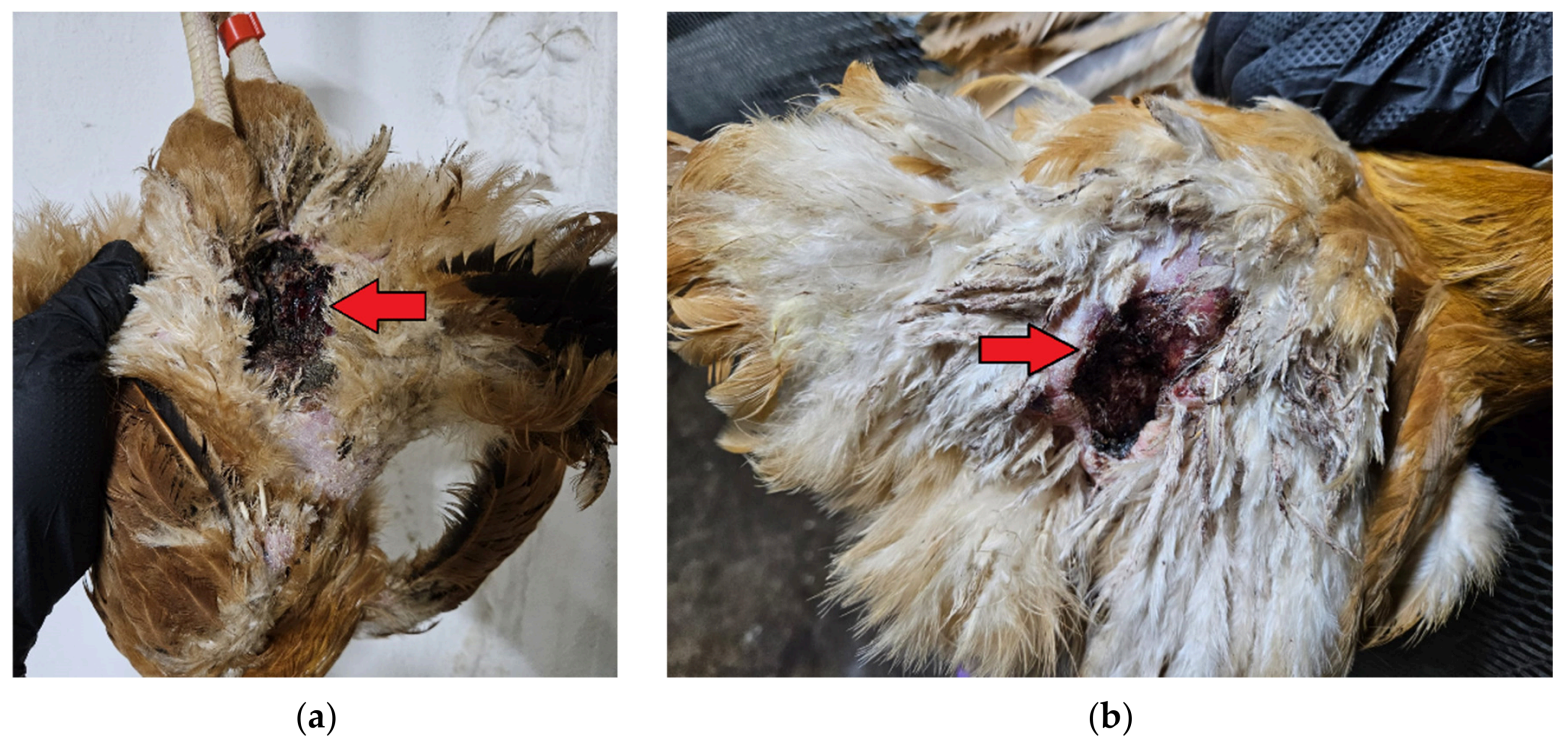
Red arrows indicate the traumatic lesions. (**a**) A wound located on the right side of the trunk, extending toward the thigh, characterized by retraction and thickening of the wound edges, with exposure of the subcutaneous and muscular tissues. The surface was necrotic and showed additional lesions consistent with cannibalistic pecking superimposed on the primary wound. (**b**) Lacerated wound located on the right lateral aspect of the trunk, caudal to the thigh, with extension onto the thigh. The lesion had an approximately elliptical outline, thickened margins, and a slightly depressed surface covered by a brownish-red crust.

**Figure 3:**
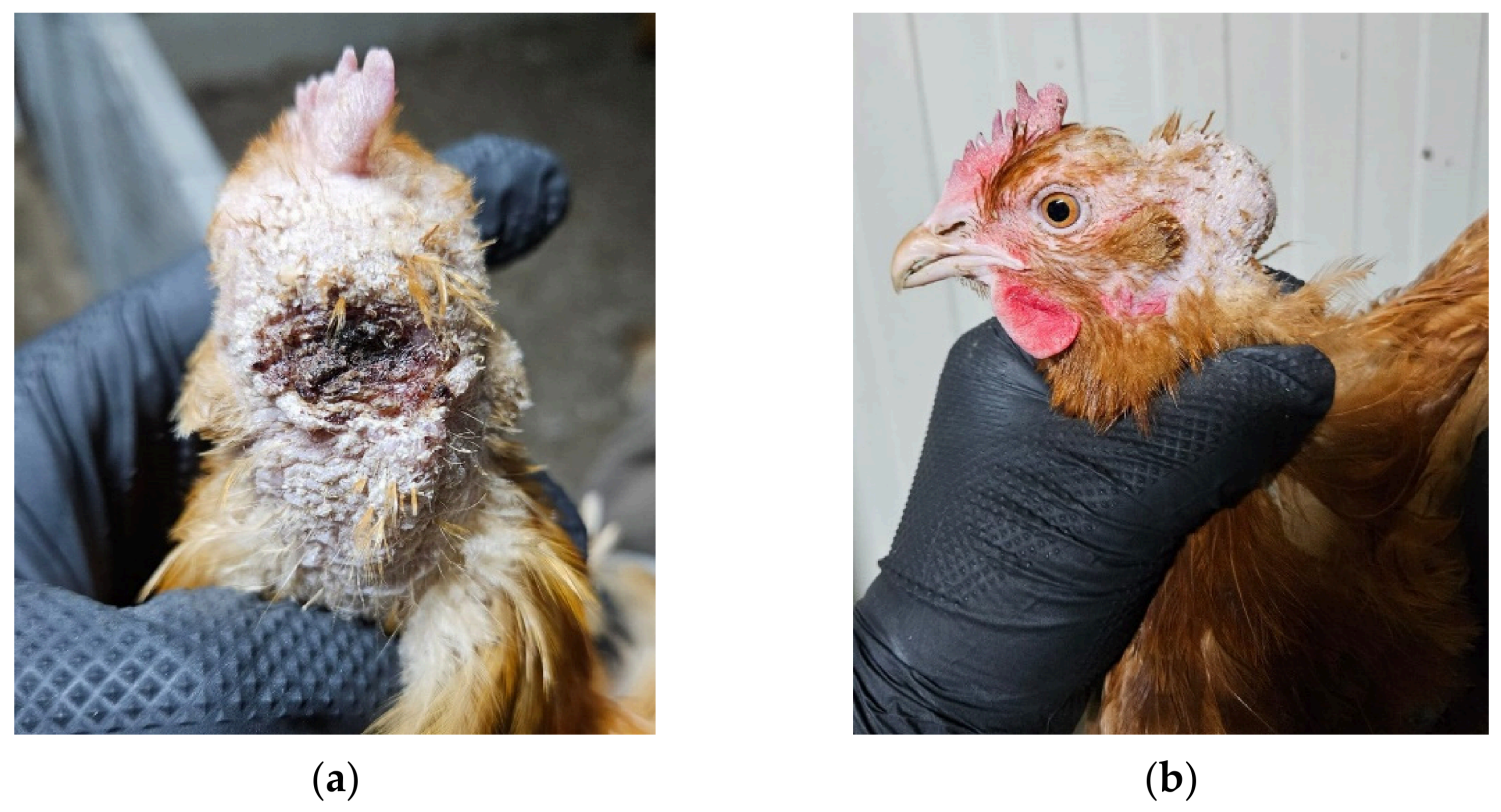
(**a**) Traumatic lesion in the dorsal region of the head, with an approximately oval outline and a surface covered by a brownish crust. The lesion showed a central depression with mildly elevated, thickened margins, resulting in a crateriform appearance. Perilesional plumage was severely compromised. (**b**) Lesion located in the occipital region, characterized by a well-defined rounded prominence suggestive of an organized abscess. The formation was firm and elastic on palpation, and plumage in the head region was severely compromised.

A single traumatic lesion was recorded in each of the 29 affected hens. Lesions were located predominantly on the trunk, with occasional extension to the thigh, in 23 of the 29 hens (79.31%) (Figure 2), whereas the remaining lesions involved the head, in 6 of the 29 hens (20.69%) (Figure 3). The estimated lesion area had a median of 12.57 cm^2^ (IQR, 6.59 to 19.84 cm^2^), with a mean of 14.62 ± 12.10 cm^2^ and a range of 0.71 to 61.48 cm^2^. The composite lesion index ranged from 0 to 4, with a mean of 2.34 ± 1.32 and a median of 2.0 (Q1 to Q3, 1.0 to 3.5). The minimum score of 0 was recorded in 3 hens (10.34%), whereas the maximum score of 4 was recorded in 6 hens (20.69%); intermediate scores of 1 to 3 were observed in the remaining 20 hens (68.97%).

### 3.2. Body Weight and Plumage Condition

Body weight was higher in control hens (2157.2 ± 136.0 g; *n* = 10; range, 1939 to 2324 g) than in affected hens (1836.2 ± 294.4 g; *n* = 29; range, 1335 to 2436 g) (Mann–Whitney *U* = 48.0, *p* = 0.002). Consistent with the group assignment criteria, control hens had uniformly intact plumage (5.00 ± 0.00), whereas affected hens had lower plumage scores (2.96 ± 0.98; Mann–Whitney *U* = 0.00, *p* < 0.001).

### 3.3. Ovarian Ultrasonographic Findings in Control Hens

**Figure 4:**
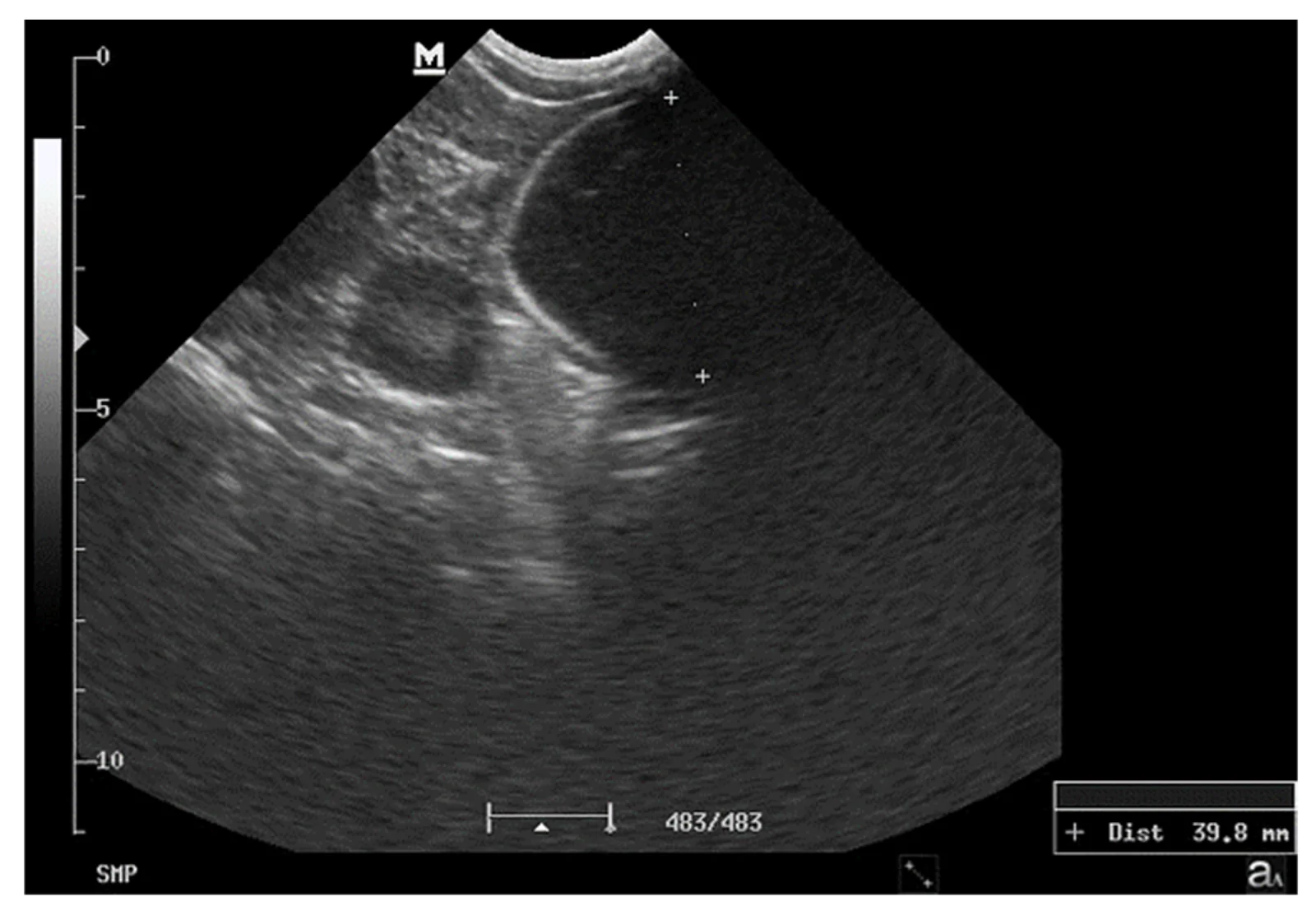
Ultrasonographic image of hen ovarian follicles.

Visible ovarian follicles were identified by ultrasonography in all control hens (*n* = 10; Figure 4). Maximum follicular diameter ranged from 33.9 to 51.3 mm, with a mean of 42.8 ± 5.6 mm, a median of 40.3 mm, and Q1 to Q3 values of 39.3 to 48.15 mm. The number of detectable follicles ranged from 3 to 7, with a mean of 5.3 ± 1.3 and a median of 5.

### 3.4. Ovarian Activity Score in Affected Hens

**Figure 5:**
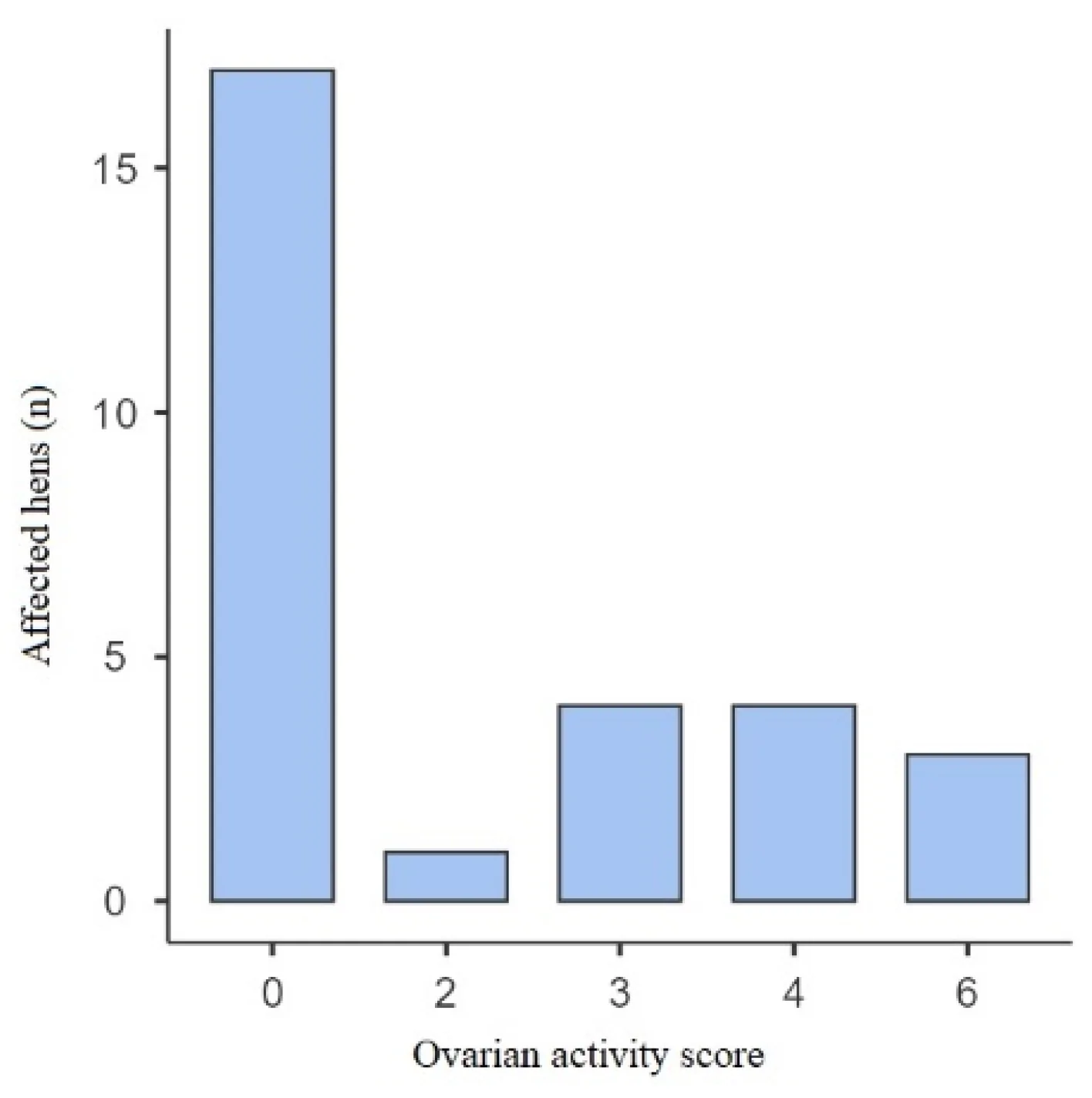
Distribution of ovarian activity scores based on ultrasonographic evaluation in the affected hens.

In affected hens (*n* = 29), the ovarian activity score showed a predominance of low values (Figure 5). A score of 0, corresponding to an inactive ovary, was recorded in 17 hens (58.62%). A score of 2 was recorded in 1 hen (3.45%), scores of 3 and 4 were each recorded in 4 hens (13.80%), and the maximum score of 6 was recorded in 3 hens (10.34%). The first quartile and median were both 0, whereas the third quartile was 3.5 (IQR, 3.5). The mean ovarian activity score was 1.65.

### 3.5. Serum Corticosterone Concentrations

**Figure 6:**
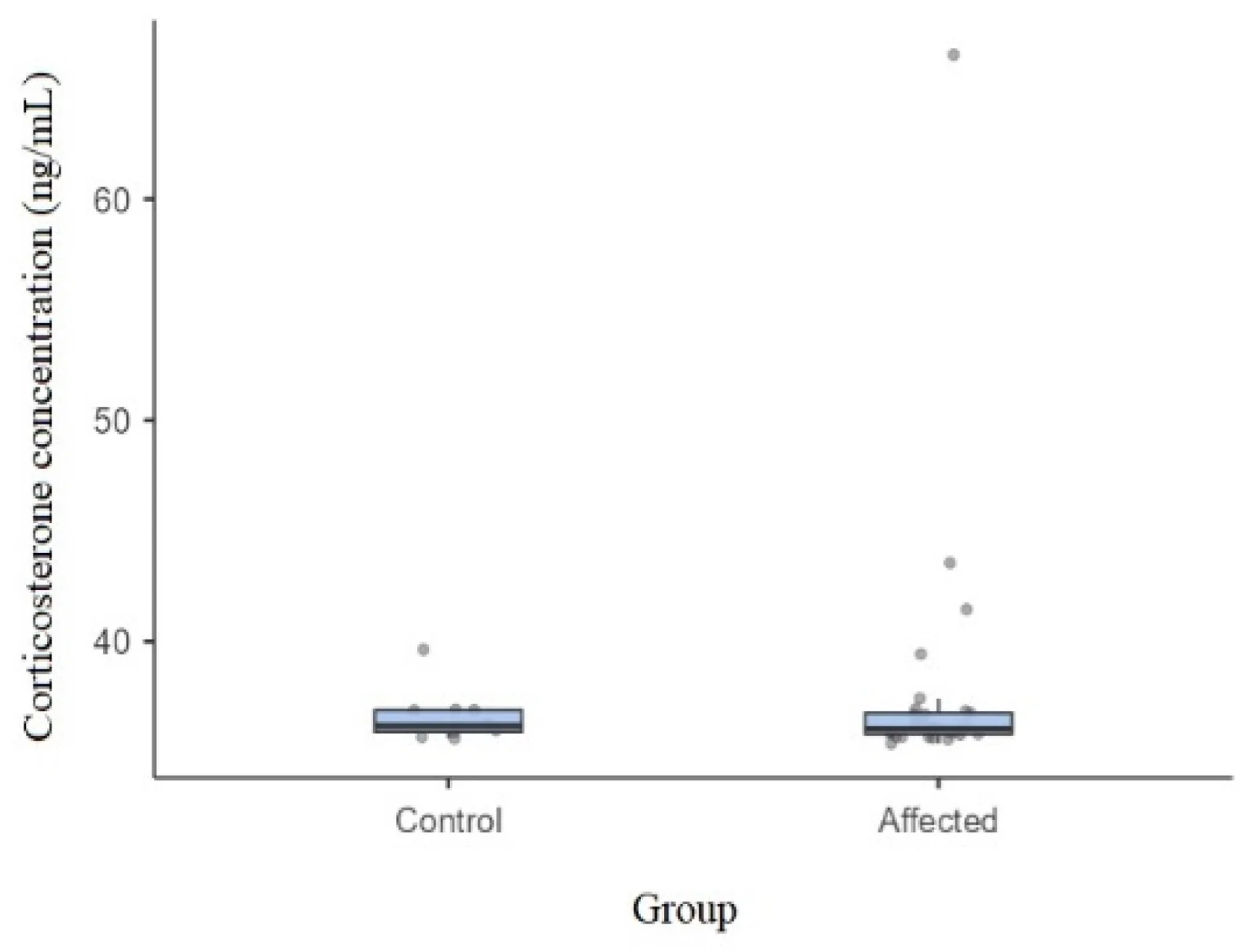
Serum corticosterone concentrations in control hens and hens with traumatic lesions.

In hens with traumatic lesions (*n* = 29), the mean serum corticosterone concentration was 37.73 ± 5.82 ng/mL, with a median of 36.06 ng/mL and a range of 35.40 to 66.52 ng/mL. In control hens (*n* = 10), the mean concentration was 36.59 ± 1.17 ng/mL, with a median of 36.19 ng/mL and a range of 35.62 to 39.64 ng/mL (Figure 6). Serum corticosterone concentrations did not differ between hens with traumatic lesions and control hens (Mann–Whitney *U* = 133, *p* = 0.711).

### 3.6. Lesion-Centered Analyses Within the Affected Group

#### 3.6.1. Associations with Traumatic Lesion Index

In affected hens (*n* = 29), the lesion index was negatively correlated with plumage score (Spearman’s ρ = −0.416, *p* = 0.025) and was weakly negatively correlated with body weight (ρ = −0.320, *p* = 0.089), whereas no association was detected with the ovarian activity score (ρ = 0.007, *p* = 0.971) or corticosterone concentration (ρ = 0.185, *p* = 0.337). Given that lower lesion-index values indicate greater lesion severity, the negative coefficients indicate that more severe lesions were associated with higher body weight and higher plumage scores.

#### 3.6.2. Associations with Traumatic Lesion Location

**Figure 7:**
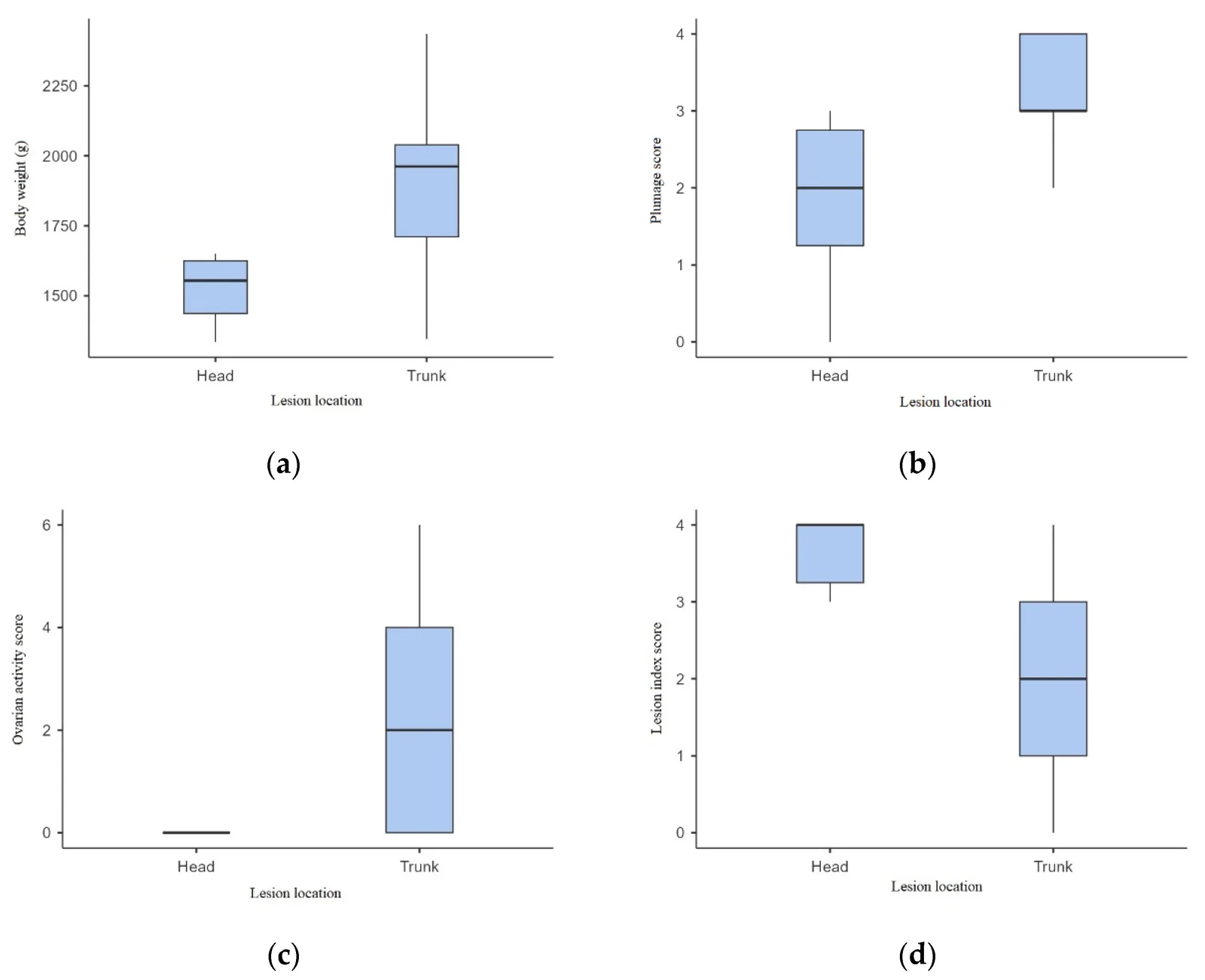
(**a**) Distribution of body weight according to lesion location. (**b**) Distribution of plumage scores according to lesion location. (**c**) Distribution of ovarian activity scores according to lesion location. (**d**) Distribution of lesion index scores according to lesion location.

Exploratory comparisons between hens with cephalic and trunk lesions indicated differences in several clinical and reproductive parameters (Figure 7). Body weight was lower in hens with cephalic lesions than in hens with trunk lesions (Mann–Whitney *U* = 13.5, *p* = 0.003). The plumage score was also lower in hens with cephalic lesions than in hens with trunk lesions (Mann–Whitney *U* = 20.0, *p* = 0.005). The ovarian activity score was lower in hens with cephalic lesions than in hens with trunk lesions (Mann–Whitney *U* = 33.0, *p* = 0.032). By contrast, the lesion index was higher in hens with cephalic lesions than in hens with trunk lesions (Mann–Whitney *U* = 17.0, *p* = 0.005), whereas corticosterone concentration did not differ according to lesion location (Mann–Whitney *U* = 53.5, *p* = 0.419).

### 3.7. Associations Among Body Weight, Plumage Condition, Ovarian Activity, and Corticosterone Within the Affected Group

In affected hens (*n* = 29), the plumage score was positively correlated with body weight (Spearman’s ρ = 0.427, *p* = 0.021), and body weight was positively correlated with the ovarian activity score (ρ = 0.467, *p* = 0.011). No association was observed between the plumage score and ovarian activity score (ρ = 0.215, *p* = 0.264). Likewise, corticosterone concentration was not associated with body weight (ρ = 0.000, *p* = 0.998), the plumage score (ρ = −0.221, *p* = 0.249), or the ovarian activity score (ρ = 0.322, *p* = 0.089).

## 4. Discussion

### 4.1. Principal Findings

The present findings indicate that traumatic lesions associated with aggressive mating in broiler breeder hens should not be viewed merely as visible sequelae of mating-associated trauma but rather as clinically relevant indicators of compromised biological status in affected females. Injured hens had significantly lower body weight than controls, and more than half of the affected birds showed ultrasonographic evidence of ovarian inactivity, suggesting that mating-related trauma may be associated with impaired somatic condition and reproductive status. Within the affected group, the positive associations of body weight with both plumage score and ovarian activity further suggest that these variables capture interconnected dimensions of overall physiological condition. Particularly noteworthy was the apparent importance of lesion topography, as hens with cephalic lesions exhibited lower body weight, poorer plumage condition, and lower ovarian activity than hens with trunk lesions, despite lesion scores indicating less severe macroscopic damage. This pattern suggests that lesion location may convey biologically meaningful information not fully reflected by gross lesion severity alone. By contrast, serum corticosterone concentrations did not differ between groups, indicating that a single circulating measure of this hormone may have limited sensitivity for detecting the broader biological burden associated with aggressive mating-related trauma.

Taken together, these findings raise the possibility of a bidirectional relationship between mating-related trauma, overall condition, and reproductive status. On the one hand, traumatic injury may contribute to deterioration in physical condition and, in some affected hens, to reduced or discontinued reproductive activity. On the other hand, hens entering mating interactions in poorer condition may be more susceptible to traumatic injury. These possibilities are not mutually exclusive and may contribute to a self-reinforcing process in which injury, declining condition, and impaired reproductive status become interconnected. Given the cross-sectional design of the present study, however, the direction of these associations cannot be established.

### 4.2. Traumatic Lesion Characteristics in Affected Hens

In the present study, affected hens generally exhibited a single traumatic lesion, most commonly located either on the dorsal head region or unilaterally on the caudolateral trunk and lateral thigh. This distribution is consistent with previous descriptions of mating-related injuries in broiler breeder hens, in which lesions have been reported predominantly on the head, trunk, and thighs and, in some cases, beneath the wings [1,4,7]. The observed topography is also biologically plausible in relation to the mechanics of forced mating, during which the male may contact the female’s head and dorsolateral body regions with the beak and claws during mounting and restraint.

A further notable feature was the predominantly focal character of the lesions. Multiple distinct traumatic lesions on the same hen were uncommon, suggesting that mating-related trauma may often begin as a single localized injury rather than as diffuse body-wide damage. From a biological standpoint, such a lesion may then follow more than one trajectory: it may remain relatively limited, consistent with subsequent avoidance of further traumatic contact, or it may become aggravated over time through repeated mating attempts and/or superimposed social pecking damage. This interpretation is consistent with earlier reports indicating that lesions initially produced during mating may later be compounded by repeated traumatic contact or by secondary social injury, including cannibalistic damage [1].

Lesions involving the trunk tended to occupy a larger surface area than those on the head, which is consistent with the broader contact surface likely generated by claw action over the body wall. At the same time, the unilateral distribution frequently observed on the trunk and thigh suggests that these lesions do not arise as random abrasions but rather reflect repeated contact applied in a more spatially constrained manner. Several factors may contribute to this pattern, including male–female size mismatch, instability during mounting, female escape behavior, and variation in claw contact during forced copulation [6,26]. Because routine toe treatment in broiler breeder males does not fully eliminate hen injury, such patterns may still emerge even in flocks in which males have undergone preventive claw or toe procedures [1].

Overall, these observations indicate that mating-related trauma in the examined hens followed a recognizable topographic and morphological pattern rather than occurring as randomly distributed skin injury. This point is important because it suggests that lesion characteristics may carry biological information beyond simple presence or size. In particular, the combination of focal onset, anatomically recurrent distribution, and occasional subsequent aggravation supports the view that traumatic lesions associated with aggressive mating are better understood as part of an evolving process than as isolated static findings.

### 4.3. Body Weight and Plumage Integrity in Affected Hens

Body weight provided an additional indicator of general biological condition in the present study. Affected hens were lighter than controls, although this difference may partly reflect the selection of control hens for intact plumage and good general clinical condition. In that context, the lower body weight of affected hens is consistent with the possibility that mating-related traumatic lesions should not be regarded solely as external findings but rather as indicators of a less favorable overall biological condition. Previous studies have shown that body weight is closely associated with overall health status and reproductive performance in hens, although it is also shaped by rearing and nutritional management [27,28,29,30,31]. Within the affected group, the positive association between plumage score and body weight further supports this interpretation, suggesting that poorer feather cover and reduced body condition tend to co-occur in injured hens. By contrast, lesion severity showed only a weak association with body weight, suggesting that lesion severity alone did not fully explain variation in body condition within the affected group. Taken together, these findings suggest that body weight is a more informative correlate of biological condition than gross lesion severity, and they further support the need to consider lesion characteristics beyond severity alone.

Plumage condition should be interpreted more cautiously in the present study. Although affected hens had lower plumage scores than controls, intact plumage was part of the control-group selection criteria, and the between-group difference, therefore, cannot be treated as an entirely independent finding. Nevertheless, plumage remained informative within the affected group itself. The positive association between plumage score and body weight suggests that feather cover and somatic condition reflect related dimensions of overall biological status. However, feather loss may also be influenced by mating activity and mechanical wear, and should therefore not be interpreted solely as a marker of body condition. Previous experimental observations in broiler breeders have shown that greater feather loss is not necessarily associated with increased mating frequency [32]. This interpretation is consistent with previous work indicating that plumage condition is a multifactorial but valuable indicator of welfare and physiological state in poultry, influenced by age, housing conditions, nutrition, health, and behavioral interactions [33,34,35]. At the same time, plumage score was not associated with ovarian activity in affected hens, suggesting that feather condition alone may not reliably reflect reproductive status under conditions of ongoing trauma. In this context, plumage integrity is best interpreted not as a direct proxy for reproductive function but as an adjunct indicator of general condition in hens exposed to mating-related injury. Within the affected group, higher lesion severity was unexpectedly associated with higher plumage scores. More broadly, the intact plumage observed in control hens may reflect not only the enrollment criteria applied at selection but also more favorable social and management-related conditions supporting the maintenance of feather cover and overall condition [26,36].

### 4.4. Ovarian Activity and Reproductive Implications

Ovarian activity was frequently reduced in affected hens, with more than half of the birds showing an ultrasonographically inactive ovary. Within this group, body weight was positively associated with ovarian activity, whereas no association was detected between ovarian activity and the lesion severity index. Together, these findings suggest that ovarian status in traumatized hens was more closely related to body condition than to the gross severity of the lesion itself. This interpretation is consistent with previous work indicating that reproductive function in hens is closely linked to body weight and condition [29,37,38].

From a discussion standpoint, the most relevant point is not the full distribution of ovarian scores but rather that ovarian inactivity was common among affected hens and that greater ovarian activity was observed in heavier birds. Ovarian regression and impaired reproductive activity may occur in the context of acute disease, injury, severe stress, or systemic infectious processes [39,40]. At the same time, because lesion severity was not associated with ovarian activity, the traumatic lesion index does not appear to function as an independent indicator of reproductive status. Thus, based on the results presented thus far, body weight appears to be a more informative correlate of ovarian activity than lesion severity. At the same time, the findings suggest that lesion location may also be an important dimension in the biological interpretation of traumatic mating-related injuries.

### 4.5. Biological Relevance of Lesion Location

Exploratory comparisons suggested that lesion location may provide additional information about biological burden beyond gross lesion severity alone. Hens with cephalic lesions had lower body weight, poorer plumage condition, and ovarian inactivity compared with hens bearing trunk lesions, despite having higher lesion index values, which in the present scoring system corresponded to less severe macroscopic damage. Thus, lesions involving the head were, on average, less severe on gross examination yet were associated with a less favorable biological profile. By contrast, corticosterone concentrations did not differ according to lesion location. Together, these findings suggest that the significance of mating-related traumatic lesions cannot be inferred from macroscopic severity alone and that lesion topography may provide biologically meaningful information beyond lesion presence and gross severity. In particular, cephalic lesions may identify a subgroup of hens in a particularly compromised biological state.

A plausible explanation for the apparent importance of cephalic lesions is that trauma in this region is not only anatomically distinct but also socially conspicuous. In domestic fowl, visible alterations in external phenotype can modify conspecific responses, and experimentally marked birds have been shown to attract increased aggression even in familiar groups [41,42,43]. Within this framework, a cephalic lesion may function not only as an initial mating-related injury but also as a visible social cue that increases the likelihood of subsequent aggression or social challenge. One possible consequence is a cascade-like process in which the initial traumatic event is followed by increased visual conspicuity, greater exposure to conspecific aggression, progressive deterioration in body condition and plumage, and, ultimately, ovarian inactivity. In this sense, lesion location may be biologically informative not because it directly determines ovarian status but because cephalic trauma may be more likely to trigger a secondary sequence of adverse social and physiological effects. However, this proposed sequence remains hypothetical, as individual behavioral interactions and changes over time were not assessed in the present study.

This interpretation also helps place the present findings within the broader literature. Existing studies on traumatic lesions associated with sexual aggression in broiler breeders have focused primarily on behavioral aspects of male–female interactions [2,3,5,6,7,11], whereas data on the longer-term biological status of injured hens remain limited. Previously reported reductions in reproductive performance have most plausibly been interpreted through behavioral mechanisms, including female avoidance of males, rather than through direct evaluation of the hen’s physiological state. The present findings extend this literature by suggesting that mating-related trauma may be associated not only with visible tissue damage but also with poorer general condition and altered reproductive status, particularly in hens with cephalic lesions. However, the comparisons between lesion locations remain exploratory.

When considered together with our previous observations that new cephalic lesions arise predominantly during the early laying period in a comparable broiler breeder population [9], the present findings suggest a more dynamic interpretation of mating-related trauma. Rather than representing static external abnormalities, such lesions may mark different stages within a broader evolving process, beginning with lesion emergence and, in at least some hens, followed by longer-term biological consequences that remain detectable later in the production cycle. At the same time, because the present study was cross-sectional, the direction of these associations cannot be established with certainty. Mating-related trauma may contribute to subsequent deterioration in condition, but it is also possible that hens in poorer condition are more susceptible to injury or less able to avoid traumatic mating interactions. Even so, the observed pattern of lower body weight, poorer plumage condition, and ovarian inactivity in hens with cephalic lesions suggests that lesion location warrants further investigation as a potentially informative feature of mating-related injuries.

### 4.6. Interpretation of the Corticosterone Findings

The inclusion of corticosterone as a study variable was based on extensive previous evidence identifying elevated circulating corticosterone as a relevant indicator of acute stress in birds [17,44]. Because corticosterone has been widely used in poultry research across diverse experimental and husbandry contexts, its assessment in the present study was intended to determine whether hens affected by mating-related lesions also exhibited an endocrine response consistent with stress.

Interpretation of corticosterone in chickens, however, requires particular caution because circulating concentrations are influenced by multiple biological and methodological factors. Acute handling and restraint are especially important confounders, as they can rapidly increase corticosterone levels. Indeed, manual immobilization or placement in cages has been shown to induce higher corticosterone concentrations than other stressors such as thirst, hunger, or heat exposure [44]. In addition, corticosterone secretion follows a clear circadian pattern. Plasma concentrations have been reported to rise significantly between 19:00 and 9:00, with values markedly higher at 9:00 than at 19:00 [45]. Similarly, De Jong et al. (2001) observed peak corticosterone concentrations approximately four hours after lights-on [46], while Hillebrecht et al. (2024) showed that the daily peak is not explained by daytime social stress but instead represents a physiological endophenotype [47]. Together, these findings indicate that corticosterone is a highly dynamic measure whose interpretation depends strongly on the timing and conditions of sampling.

This complexity is further reflected in the broad range of corticosterone values reported in the poultry literature. Depending on methodology, environmental conditions, and experimental context, physiological concentrations in hens have been reported to range from 0.3 to 33.38 ng/mL, whereas under pronounced stress, they may range from 0.25 to approximately 150 ng/mL [17]. Such variability emphasizes that corticosterone should not be interpreted as a standalone indicator but rather in relation to the broader biological and experimental framework.

Within this context, the corticosterone concentrations recorded in the present study were slightly above the upper limit of the physiological range reported in the literature yet remained well below values typically associated with marked stress. Although corticosterone did not differ between affected and control hens and showed no association with body weight, plumage score, ovarian activity, lesion index, or lesion location, the greater dispersion of values in affected hens could indicate that traumatic lesions are associated with a subtle stress response in a subset of individuals. This interpretation should be viewed cautiously and requires confirmation in further studies.

These results indicate that corticosterone was not a discriminating variable in the present dataset. Rather than suggesting the absence of biological burden in affected hens, this finding more likely indicates that a single corticosterone measurement had limited explanatory value under the conditions of this study. By contrast, body weight, ovarian status, and lesion location differentiated affected hens more clearly than corticosterone. This pattern supports the interpretation that the consequences of mating-related trauma may be better captured by integrated changes in condition and reproductive function than by a single stress marker. This interpretation is consistent with the broader poultry literature, which highlights substantial methodological and biological variability in corticosterone measurement [17]. Taken together, these considerations suggest that multiple complementary stress-related parameters may be more informative than corticosterone alone.

### 4.7. Practical Implications for Welfare Assessment and Flock Management

The present findings may also have practical relevance for welfare assessment and flock management in broiler breeder flocks. Previous studies have raised concerns that aggressive mating may adversely affect female welfare [4,5,6], and the current results extend this perspective by suggesting that at least some mating-related traumatic lesions, particularly those involving the head, may be associated with a more systemic state of biological compromise. When considered together with our previous observations that cephalic lesions arise predominantly during the early laying period [9], the present findings support a more dynamic interpretation of these lesions, in which lesion emergence represents only one stage of a broader process whose consequences may remain detectable later in the production cycle. Although neither welfare nor productive performance was measured directly, the results indicate that lesion assessment in commercial breeder flocks may benefit from considering anatomical location, particularly cephalic involvement, in addition to lesion presence and gross severity. Such an approach may improve the identification of hens requiring closer monitoring or management attention under field conditions.

### 4.8. Study Limitations and Future Directions

Several limitations should be considered when interpreting the present findings. First, the study was cross-sectional and based on a single assessment performed at 40 weeks of age, which does not allow the temporal direction of the observed associations to be established with certainty. Mating-related trauma may contribute to subsequent deterioration in condition, but it is also possible that hens in poorer condition are more susceptible to injury or less able to avoid traumatic mating interactions. Second, the study was conducted in a single commercial flock of one slow-growing broiler breeder genotype, and the number of enrolled hens was limited, particularly in the control group; therefore, caution is warranted when extrapolating these findings to other flocks, management systems, or genetic backgrounds. Furthermore, control hens were selected for intact plumage and good general clinical condition, in addition to the absence of traumatic lesions. These selection criteria may have introduced bias into between-group comparisons of general biological condition, including body weight, as observed differences may partly reflect the selection criteria rather than associations with traumatic lesions alone. In addition, affected hens represented a sampled subset of injured birds present at the time of evaluation rather than the entire affected population. Comparisons between lesion-location categories were exploratory and limited by the small number of hens with cephalic lesions (*n* = 6). The absence of adjustment for multiple testing increases the risk of false-positive findings, and these results require confirmation in larger samples. Third, ovarian activity was assessed ultrasonographically as an indicator of reproductive status, whereas direct reproductive outcomes such as egg production, fertility, or hatchability were not measured. Similarly, welfare and productive performance were not evaluated directly, and the present results should therefore be interpreted in terms of biological status and practical relevance rather than demonstrated effects on welfare or productivity. Finally, the composite lesion index and ovarian activity score were study-specific constructs designed for internal comparison, serum corticosterone was assessed from a single blood sample under field conditions, and individual behavioral interactions were not recorded, which limits both mechanistic interpretation and the precision of stress-related assessment.

Future studies should address these limitations by using longitudinal designs that follow individual hens from the onset of lay through the later stages of the production cycle. Such approaches would help clarify whether cephalic lesions precede subsequent deterioration in condition and ovarian activity or instead identify hens that are already more vulnerable to traumatic mating. Integrating repeated lesion assessment with behavioral observations, direct reproductive outcomes, and additional physiological indicators would further improve understanding of the biological significance of mating-related trauma. More broadly, replication across multiple flocks and genetic backgrounds would be valuable to determine the generality of the present findings and to evaluate whether lesion topography can be used as a practical indicator for targeted monitoring under commercial conditions.

## 5. Conclusions

Mating-related traumatic lesions in slow-growing broiler breeder hens were associated with poorer biological condition under commercial conditions. Affected hens had lower body weight than lesion-free controls, and ultrasonographically detectable ovarian inactivity was common among injured birds. Within the affected group, body weight was positively associated with both plumage condition and ovarian activity, suggesting that these variables reflect related dimensions of overall biological status. Gross lesion severity alone did not appear to be an informative indicator of broader biological compromise, whereas exploratory comparisons suggested that lesion location may provide additional biological information beyond macroscopic severity alone. In particular, hens with cephalic lesions showed lower body weight, poorer plumage condition, and ultrasonographically detectable ovarian inactivity despite having less severe lesions on gross examination. Serum corticosterone measurement alone may not be sufficient to assess stress levels in hens with traumatic lesions associated with aggressive mating.

## Data Availability

The original contributions presented in this study are included in the article. Further inquiries can be directed to the corresponding author.
